# Investigating Risk Factors for Laryngotracheal Reconstruction and Assessing Postoperative Management

**DOI:** 10.7759/cureus.78001

**Published:** 2025-01-26

**Authors:** Raj Patel, Caleb Keng, Tadas Masys, Shareef Amor, Jay Patel

**Affiliations:** 1 Otolaryngology - Head and Neck Surgery, Loyola University Chicago Stritch School of Medicine, Maywood, USA; 2 Biology, Loyola University of Chicago, Chicago, USA; 3 Dentistry, Roseman University of Health Sciences College of Dental Medicine, South Jordan, USA; 4 Dentistry, Aegis Dental Group, Warsaw, USA

**Keywords:** demographics, etiology, laryngotracheal reconstruction, outcome measure, risk factors

## Abstract

This study examines demographic and etiological factors influencing the need for laryngotracheal reconstruction (LTR) by analyzing clinical studies with clear outcome measures, pre- and post-treatment scores, and demographic data while excluding case reports, reviews, and abstracts. A literature search through PubMed and Embase identified 49 articles, which were narrowed to six after abstract analysis. The results indicate that sex, age, and ethnicity were not significant predictors of the need for LTR, whereas the underlying etiology (particularly trauma, prolonged intubation, and idiopathic causes) significantly influenced the necessity for surgery and surgical outcomes. Furthermore, postoperative rehabilitation emerged as a critical factor in improving patient recovery, particularly for regaining swallowing function and alleviating dysphagia. These findings underscore the importance of tailoring surgical approaches based on etiology and highlight the essential role of rehabilitation in optimizing recovery following LTR.

## Introduction and background

Laryngotracheal reconstruction (LTR) is a surgical procedure designed to widen a narrowed airway and restore essential function. By establishing a stable, clear, and permanent airway, LTR aims to eliminate the necessity of artificial airways such as endotracheal tubes or tracheostomy while simultaneously preserving or optimizing voice quality and swallowing capabilities. The procedure is most often indicated in cases of laryngotracheal stenosis (LTS) and involves surgically opening the stenosed segment and placing cartilage grafts, frequently harvested from costal or thyroid cartilage, to increase the diameter of the laryngotracheal area [1].

Surgical approaches to LTR require a comprehensive understanding of the involved anatomical structures. The oropharynx, a shared passageway for both air and food, is critical in coordinating swallowing and respiration. The epiglottis deflects solids and liquids traveling through the oropharynx from entering the larynx, directing them down the esophagus and protecting the lower airways during swallowing [2]. The larynx plays a multifaceted role in airway movement, respiration, and voice production by housing the vocal folds, which regulate air passage and produce sound through vibratory motion. Its anatomy, including layered vocal fold structures and precise muscular control, ensures effective phonation, airway movement, and respiratory function [3].

**Figure 1:**
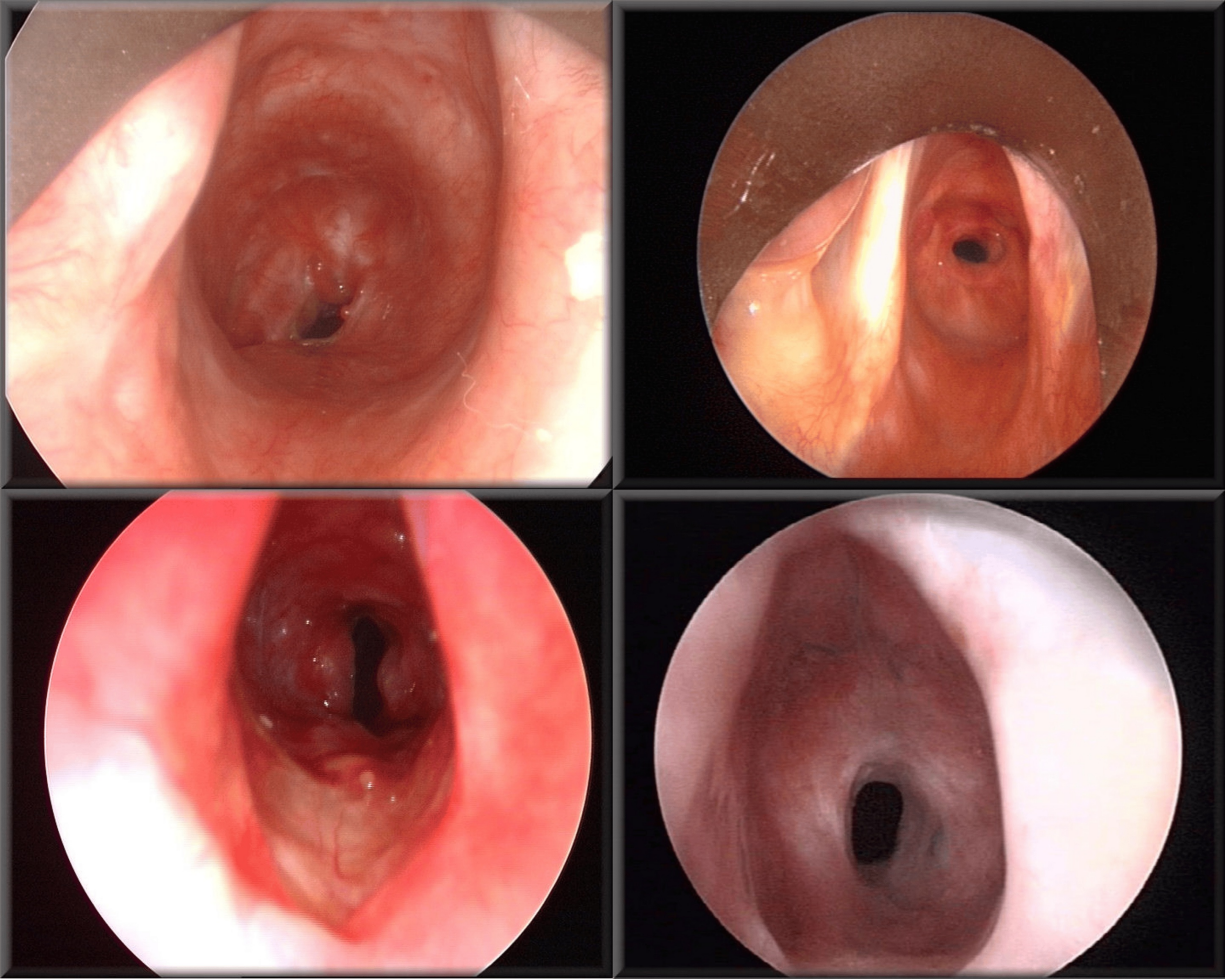
Phenotypic examples of laryngotracheal stenosis. Reproduced with permission from [4].

LTS involves significant anatomical changes that affect airway dynamics and function. Stenosis and airway collapse most commonly occur below the vocal cords in the subglottic region, where the airway narrows due to excessive scar tissue formation and structural alterations in the cartilaginous and soft tissue components (Figure 1) [4]. The resultant narrowing creates a flow-limiting segment, particularly during expiration, leading to turbulent airflow and increased airway resistance. This altered airflow pattern results in significant energy loss and can create recirculation zones downstream of the obstruction like the trachea, further complicating airway dynamics [5]. The collapse affects not only breathing but also impacts voice production and could interfere with normal swallowing mechanisms at the pharynx, epiglottis, and esophagus.

Multiple etiological pathways contribute to LTS. The formation of scar tissue is frequently associated with prolonged intubation or tracheostomy, but LTS can also arise from congenital abnormalities, autoimmune diseases, tumors/masses, blunt laryngeal trauma, and idiopathic causes [6]. Less commonly, inhalational chemical injuries or gastroesophageal reflux disease (GERD) may also contribute to the development of laryngotracheal stenosis [7].

The choice of laryngotracheal reconstruction technique is determined by carefully evaluating multiple factors: the location, extent, and severity of the stenosis, the patient's specific airway collapse pattern, overall health status, and risk of complications. LTR can be delivered either endoscopically or through open-airway surgery, with each approach offering distinct advantages based on the clinical presentation.

Endoscopic LTR is a minimally invasive approach that avoids external incisions. The airway is accessed using a laryngoscope equipped with a camera, light source, and surgical instruments. This technique can be used either to place grafts during laryngotracheoplasty or to relieve airway narrowing through alternative methods like lasers or balloon dilation, eliminating the need for a full reconstruction. While endoscopic LTR offers a less invasive option, it may not be suitable for patients with severe airway narrowing or extensive scarring [8].

Open-airway LTR, a procedure that involves an incision on the neck, can be performed as either a single-stage or double-stage procedure. In single-stage LTR, after the airway is widened with precisely shaped cartilage grafts, an endotracheal tube is placed to stent the reconstructed area, remaining in place for several days to about two weeks depending on healing progression and graft characteristics. Any existing tracheostomy is typically removed during this procedure. In double-stage LTR, while the basic widening technique remains similar to a single-stage LTR, either a tracheostomy tube is maintained below the reconstruction site, or a stent is placed around the grafts. This supporting framework remains in place for approximately four to six weeks for healing, after which it is removed in a separate surgical procedure [9,10]. Endoscopic and single-stage open-airway procedures generally are recommended for mild cases of stenosis. For more severe cases of stenosis, particularly in the presence of significant comorbidities that may complicate surgery such as heart, lung, and neurological conditions, a double-stage procedure is often recommended [11]. Sometimes, the narrow part of the airway is removed completely, and the remaining segments are sewn together, which is known as a resection [12].

Hybrid laryngotracheal reconstruction, also known as “one-and-a-half-stage reconstruction,” represents a middle ground between single and double-stage procedures and was first pioneered on pediatric patients [13]. In this technique, two stents are used simultaneously: a long stent is inserted through the existing tracheostomy tube, while a second, smaller stent is placed through the opening in the trachea. This dual-stent approach ensures the patient maintains a secure secondary airway both during and after surgery [14].

Current literature provides substantial insights into the risk factors associated with decannulation failure, which is defined as the inability to breathe following the removal of a tracheostomy tube [15,16]. In cases of decannulation failure, LTR may be considered a corrective surgical option. Identified risk factors for decannulation failure include diabetes, GERD, and grade 4 airway stenosis [15]. While these factors highlight the patients most vulnerable to decannulation failure, there is limited demographic data addressing risk factors specific to laryngotracheal failure (LTF) in general. Existing evidence suggests that patients with severe stenosis and a higher Charlson Comorbidity Index (CCI) are at an increased risk of requiring LTR [16].

The purpose of this systematic review is to synthesize the current body of research on LTR approaches, focusing particularly on identifying any demographic and etiological factors that may predispose patients to LTF and subsequent reconstruction. Because LTR constitutes a relatively small fraction of otolaryngology procedures (fewer than 3% of ENT surgeries), many existing studies feature small patient sample sizes [17]. Consequently, one of the aims of this review is to determine whether demographic characteristics, including age, sex, and ethnicity, along with etiological factors in these studies significantly influence the need for LTR. Additionally, this review explores the various surgical approaches currently employed, aiming to identify if there is an optimal technique based on patient-specific risk factors and etiology. A deeper understanding of these factors could enhance patient outcomes by facilitating tailored LTR approaches.

## Review

Methods 

**Figure 2:**
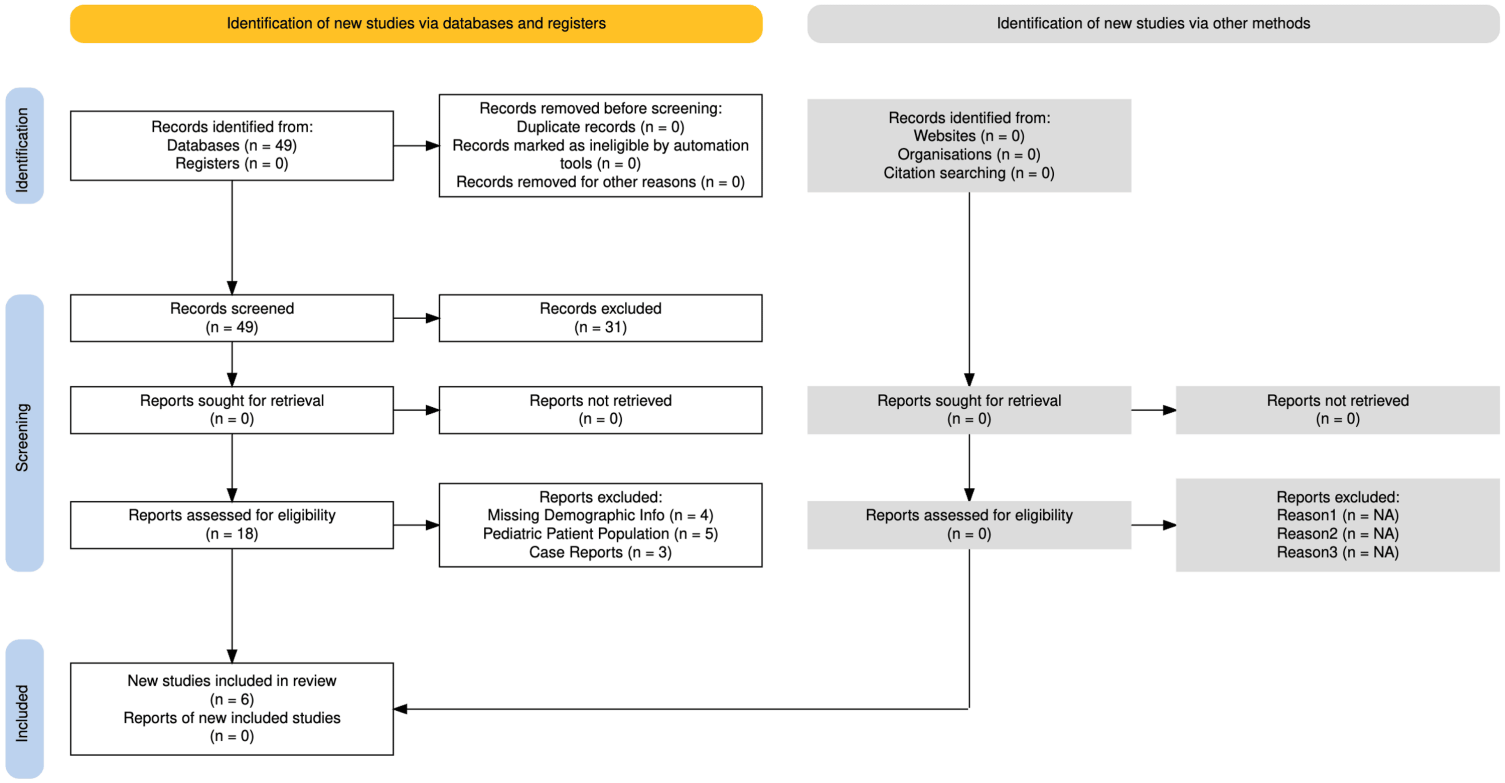
PRISMA flowchart. PRISMA: Preferred Reporting Items for Systematic Reviews and Meta-Analyses.

For the development of this systematic review, a comprehensive literature search was conducted using Medline (PubMed) and Embase to identify clinical studies published between December 2014 and December 2024. Given the rarity of laryngotracheal reconstruction, 49 articles were initially identified. The inclusion criteria were as follows: studies involving adult patients who underwent laryngotracheal reconstruction due to airway stenosis, studies that included demographic information, and those that reported on etiological factors and/or outcome measures. If a study provided detailed demographic and etiological data but lacked outcome measures, it was still included due to the focus of this review on risk factors. Studies were excluded if they lacked demographic or etiological information, if they did not specify whether patients underwent reconstructive surgery for stenosis, or if they involved pediatric patients. The reason why this paper excluded the pediatric patient population is due to the possibility that surgical approaches may differ due to a difference in anatomical maturity, which may render different surgical approaches and outcomes. Additionally, case reports, discussion panels, literature reviews, meta-analyses, and abstracts were excluded from the review. The team systematically screened and included articles based on the pre-established criteria, as depicted in the PRISMA (Preferred Reporting Items for Systematic Reviews and Meta-Analyses) flowchart (Figure 2).

**Figure 3:**
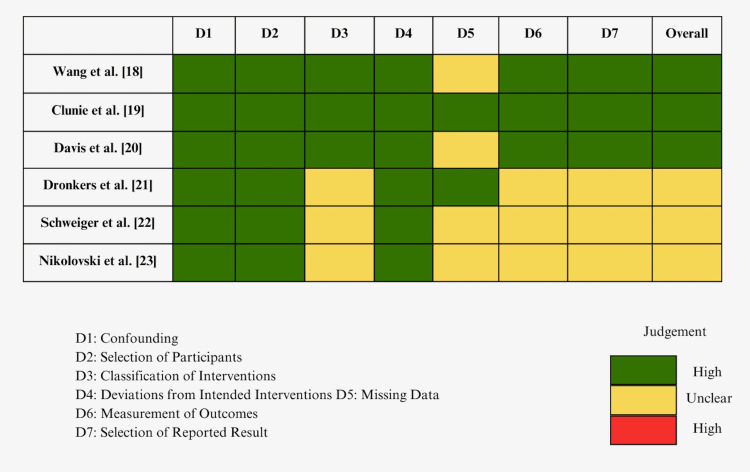
Cochrane risk-of-bias assessment.

Afterward, a Cochrane risk-of-bias assessment was done for the individual studies, and overall bias alongside the criteria assessed is showcased in Figure 3. After selecting the final included studies, the team compiled them into a table and gathered data to compare the results (Table 1).

**Table 1:** Demographic and etiology information extraction. LTR: laryngotracheal reconstruction.

Article	Median age (years)	Gender distribution	Ethnicity breakdown	Causes of LTR
Wang et al. [18]	42.78 ± 16.03	Female: 20 (52.6%), Male: 18 (47.4%)	N/A	Intubation/ventilation: 12 (31.6%), Trauma: 10 (26.3%), Idiopathic: 5 (13.2%), Laryngeal cancer surgery: 8 (21.0%), Thyroid cancer surgery: 3 (7.9%)
Clunie et al. [19]	46 (34–58)	Female: 10 (50%), Male: 10 (50%)	Asian – any other: 2 (10%), Black – any other: 1 (5%), White British: 15 (75%), White – any other: 2 (10%)	Intubation/Tracheostomy: 11 (55%), Autoimmune: 6 (30%), Trauma: 2 (10%), Idiopathic: 2 (10%)
Davis et al. [20]	51 (39–65)	Female: 8 (89%), Male: 1 (11%)	N/A	Idiopathic: 9 (100%)
Dronkers et al. [21]	45.8 ± 16.2	Male: 26 (60.5%), Female: 17 (39.5%)	White: 24 (55.8%), Black: 12 (27.9%), Asian: 4 (9.3%)	Trauma: 6 (14%), Prolonged intubation: 30 (69.8%), Tracheostomy: 7 (16.3%)
Schweiger et al. [22]	53.5	Female: 65 (60.2%), Male: 43 (39.8%)	N/A	Thyroid cancer: 10 (9.3%), Tracheal cancer: 10 (9.3%), Lung cancer: 2 (1.9%), Benign disease: 85 (78.7%), Acquired stenosis: 33 (30.6%), Idiopathic: 28 (26.0%), Malacia: 9 (8.3%), Wegener’s disease: 7 (6.5%), Other: 7 (6.5%)
Nikolovski et al. [23]	N/A	Male: 24 (66.7%), Female: 12 (33.3%	N/A	Post-polytraumatic: 24 (66%), Post-combustion: 4 (11%), Post-suicidal: 3 (8%), Congenital: 2 (5%), Idiopathic: 2 (5%), Inflammatory: 1 (2%), Post-intubation: 10 (27%), Post-tracheostomy: 14 (38%)

Results 

Analysis of gender distribution patterns among LTR patients was conducted across six studies, encompassing a total of 254 subjects (132 female individuals and 122 male individuals) (Table 2). Variability in sex ratios was observed among the studies, with male representation exhibiting a range of 11.0% to 66.7%, while female representation ranged from 33.3% to 89.0%. A chi-square analysis revealed no significant association between gender and study distribution ( p = 0.53). This indicates no statistically significant difference in gender distribution across the studies (p > 0.05), suggesting no major gender imbalance in the populations studied. 

**Table 2:** Sex distribution among laryngotracheal reconstruction subjects.

Study	Male (%)	Female (%)	Total patients (N)
Wang et al. [18]	52.60%	47.40%	38
Clunie et al. [19]	50%	50%	20
Davis et al. [20]	11%	89%	9
Dronkers et al. [21]	61%	40%	43
Schweiger et al. [22]	40%	60%	108
Nikolovski et al. [23]	66.7	33.3	36

Analysis of patient age in LTR procedures was conducted across six studies, with reported median ages spanning 42.78 to 53.5 years (Table 3). Analysis using the Kruskal-Wallis test revealed no statistically significant difference in the age distribution among the study cohorts (p = 0.41) and the absence of statistically meaningful (p > 0.05) inter-study variation in patient age. This implies that age is not a significant factor influencing the type or indication of LTR in the included studies.

**Table 3:** Median age distribution among laryngotracheal reconstruction subjects.

Study	Median age
Wang et al. [18]	42.78
Clunie et al. [19]	46
Davis et al. [20]	51
Dronkers et al. [21]	45.8
Schweiger et al. [22]	53.5
Nikolovski et al. [23]	43.6

The ethnicity distribution of patients undergoing LTR was analyzed in two of the six studies (Table 4). With a total of 61 patients across both studies, the majority of patients were White (67.2%), followed by Black (23.0%), and Asian (9.8%), with no Hispanic or Latino representation. The chi-square analysis value was 3.3, which is less than the critical value of 5.99. Because we failed to reject the null hypothesis, this indicates no statistically significant difference in ethnicity between the two studies analyzed, suggesting that ethnicity does not vary significantly across the patient populations studied for LTR. It is important to consider that the limited number of studies analyzed and the absence of certain ethnic groups warrant consideration in the interpretation of these results.

**Table 4:** Ethnicity distribution among laryngotracheal reconstruction subjects.

Group	Clunie et al. [19]	Dronkers et al. [21]	Total
White	17	24	41
Black	2	12	14
Asian	2	4	6
Hispanic or Latino	0	0	0
Total	21	40	61

All six studies were analyzed for their etiology distribution of patients undergoing LTR (Table 5). With a total of 358 patients, the analysis revealed prolonged intubation as the predominant etiological factor (17.6%), followed by trauma (15.1%), and idiopathic causes (12.8%), with non-specifically-classified etiologies comprising the majority (54.5%). A chi-squared analysis revealed significant heterogeneity in etiological distribution patterns across the patients investigated (2 = 29.68, p < 0.01). There is a highly significant variation (p < 0.001) between the studies in the distribution of the etiologies necessitating LTR.

**Table 5:** Etiology distribution among laryngotracheal reconstruction subjects.

Study	Intubation	Trauma	Idiopathic	Other	Total
Wang et al. [18]	12	10	5	11	38
Clunie et al. [19]	11	2	2	0	15
Davis et al. [20]	0	0	9	0	9
Dronkers et al. [21]	30	6	0	7	43
Schweiger et al. [22]	0	5	28	160	193
Nikolovski et al. [23]	10	31	2	17	60
Total	63	54	46	195	358

The Eating Assessment Tool (EAT)-10 is a dysphagia screening tool designed to evaluate swallowing function in patients with impaired laryngotracheal function. The scale ranges from 0 (no difficulty) to 5 (severe difficulty). Two studies that utilized the EAT-10 to measure outcomes were those by Clunie et al. and Davis et al. (Table 6) [19,20]. Both studies assessed patients in the pre-operative phase, with Clunie et al. reporting a mean dysphagia score of 1.9 and Davis et al. reporting a score of 0 (with a standard deviation of 4.56) [19,20]. Following reconstructive surgery, both studies found an increase in EAT-10 scores, with Clunie et al. reporting an increase of 1.1 and Davis et al. an increase of 1.5 (Table 6) [19,20]. Another tool used to assess laryngotracheal function is the swallow test, which measures the time it takes for a patient to swallow water. In their study, Clunie et al. stratified their patient population by gender [19]. The results showed that male individuals experienced a slight increase in swallow time of 0.1 seconds, while female individuals exhibited a small decrease of 0.1 seconds (Table 6).

**Table 6:** Pre- and post-operative outcome measures for laryngotracheal reconstruction. EAT-10: Eating Assessment Tool-10.

EAT-10			
Study	Pre-operation	Post-operation	∆
Clunie et al. [19]	1.9	3	+1.1
Davis et al. [20]	0+/-4.56	1.5+/-2.89	+1.5
Swallow test (s)			
Clunie et al. [19]	Male: 1.2 (0.9-1.3) Female: 1.6 (1-2.3)	Male: 1.3 (1.2-1.6) Female: 1.5 (1.3-1.6)	Male: +0.1 Female: -0.1

Although the primary goal of laryngotracheal reconstruction is to widen a narrowed airway and restore essential function, patients may also benefit from rehabilitation aimed at exercising the laryngotracheal structures to further regain function [1,18]. Wang et al. conducted a study in which patients underwent rehabilitation following laryngotracheal reconstructive surgery [18]. Both the EAT-10 and the swallow test were used as outcome measures. The results showed a statistically significant improvement in dysphagia, as measured by the EAT-10 (p < 0.01) after rehabilitation (Table 7). Additionally, the swallow test demonstrated significant improvements in swallow time for both water and soft food (p < 0.01), as shown in Table 7.

**Table 7:** Pre- and post-rehabilitation on laryngotracheal function. EAT-10: Eating Assessment Tool-10.

EAT-10			
Study	Pre-intervention	Post-intervention	p-value
Wang et al. [18]	11.63 +/- 3.83	2 +/- 1.18	p < 0.01
Swallow test (s)			
Water	4.68 +/- 1.45	2.05 +/- 0.61	p < 0.01
Soft food	6.21 +/- 2.99	2.21 +/- 0.62	p < 0.01

Discussion 

The primary objective of this investigation was to identify demographic and etiological factors associated with the need for laryngotracheal reconstruction (LTR) across a range of clinical studies. The analysis focused on how sex, age, ethnicity, and the underlying cause of laryngotracheal stenosis might influence the likelihood of requiring surgical reconstruction, as well as the outcomes observed in different patient populations. The findings obtained from pooled data and statistical evaluations provide several insights into the risk factors driving LTR.

The balance of male and female patients across the studies was nearly 1:1 (Male: 122, Female: 132), indicating no statistically significant difference between sexes in the requirement for LTR (Table 2). This result aligns with earlier findings in the literature, suggesting that laryngotracheal stenosis requiring reconstruction does not show a strong sex bias and can arise in patients of any sex across a broad age range [16]. Although variations did emerge in individual cohorts, such as the higher proportions of male patients in Wang et al. (52.6%) and Nikolovski et al. (66.7%), compared to the predominantly female population in Davis et al. (89%), these differences did not reach statistical significance when the data were pooled [18,20,23]. These observations imply that sex is not a principal factor guiding the decision for surgical intervention or its outcomes and that other clinical or etiological variables are likely more determinative.

Median ages among the different studies ranged from 42.78 to 53.5 years, yielding no significant age-related difference in the need for LTR (Table 3). This lack of a pronounced age-related influence suggests that laryngotracheal stenosis requiring reconstruction spans a diverse patient population, including both younger and older adults. While certain individual studies and anecdotal clinical observations have noted age-related differences in aspects such as postsurgical recovery, complication rates, or the presence of comorbidities, the pooled results here did not demonstrate a clear correlation between age and the need for LTR [18-23]. However, it remains plausible that older adults might be somewhat more prone to laryngotracheal stenosis due to factors such as prolonged intubation or the cumulative effect of multiple comorbid conditions, even if age alone is not a statistically significant predictor of surgical necessity [16,18-23].

An evaluation of ethnic background revealed no marked differences in ethnicity distributions among the study populations (Table 4). This finding suggests that the need for LTR is not primarily driven by a patient’s racial or ethnic background. However, the representation of ethnic groups was uneven, with White patients predominating in some studies (e.g., 81% in Clunie et al. and 60% in Dronkers et al.) and comparatively fewer Black, Asian, and Hispanic individuals [19,21]. The limited diversity in certain cohorts underscores the possibility that socioeconomic or healthcare access factors, often correlated with ethnicity, might not have been fully captured. Furthermore, each study took place at a distinct medical institution, which can influence the composition of patient populations, referral patterns, and the range of surgical interventions offered, thereby shaping the demographic and clinical profiles seen in each cohort. Future research that includes more ethnically diverse populations across multiple healthcare settings could provide a clearer perspective on how environmental exposures, differences in healthcare utilization, and other social determinants of health may influence the incidence and progression of laryngotracheal stenosis.

The most pronounced finding involved the underlying causes of laryngotracheal stenosis (Table 5), which varied considerably among the included studies. Trauma (including external neck injury) and prolonged intubation featured prominently in several cohorts, such as Wang et al., Clunie et al., Dronkers et al., and Nikolovski et al., whereas idiopathic factors were more prevalent in others, including Schweiger et al. (Table 5) [18-23]. Trauma-related stenosis reached notably high levels in Dronkers et al., accounting for 69.8% of cases, highlighting the impact of physical injuries among more active populations [21]. Conversely, idiopathic cases constituted 28.9% of patients in Schweiger et al., underscoring the potential roles of autoimmune processes, chronic conditions, and other less clearly defined pathophysiological mechanisms in the development of stenosis [22]. Elevated rates of intubation-related stenosis in several studies further emphasize the significance of critical care exposures, particularly repeated or prolonged intubation, in shaping the risk for laryngotracheal stenosis. 

The wide variability in etiology implies that demographic factors such as sex, age, and ethnicity may be less influential than the specific cause of stenosis when predicting the need for LTR. In clinical practice, distinct etiological pathways, such as those related to post-intubation, trauma-induced, or idiopathic causes, can lead to differing disease courses, prognoses, and responses to surgical intervention. For instance, patients with post-intubation stenosis may require a different approach to perioperative care than those presenting with acute trauma-related lesions. Identifying and characterizing these etiological differences is therefore essential for refining patient selection criteria and optimizing surgical approaches in LTR. Additionally, each study employed a different surgical technique or approach for reconstruction (Table 1). Given that significant etiological differences were observed across these studies, the underlying cause of stenosis appears closely tied to the surgical strategy selected. Aligning the surgical plan with the specific etiology may help optimize postoperative outcomes and shape subsequent treatment. Further studies could strengthen these observations by detailing the exact surgical methods used, while also incorporating standardized outcome measures to more accurately compare the effectiveness and safety of different reconstruction techniques. 

Collecting outcome measures is crucial for evaluating the efficacy of surgical reconstruction by assessing improvements in patients' symptoms. Among the seven extracted articles, three incorporated such measures. Clunie et al. and Davis et al. both employed the EAT-10 swallowing screening tool, with findings indicating an increase in dysphagia symptoms following surgery (Table 6) [19,20]. Additionally, Davis et al. reported prolonged swallow times in male patients, consistent with the observed increase in dysphagia (Table 6) [20]. While these results might suggest that laryngotracheal reconstruction yields unfavorable outcomes, it is important to recognize that rehabilitation is a standard component of postoperative care in reconstructive surgery [24]. The goal of rehabilitation is to increase the somatic and visceral function of the laryngotracheal muscles, which may be temporarily compromised by the manipulation, replacement, and excision of anatomical structures. Consequently, patients often require a period of recuperation to regain baseline function, underscoring the need for long-term follow-up measures to fully capture the benefits of LTR [24]. 

Wang et al. reported on patients who underwent T-implantation laryngotracheal surgery and then enrolled in a structured rehabilitation program aimed at restoring laryngeal and tracheal functionality [18]. Postoperative assessments revealed statistically significant improvements in patients’ swallowing capabilities, eating function, and dysphagia symptoms following rehabilitation (Table 7). This finding suggests the importance of incorporating rehabilitation into the postoperative treatment plan to address the functional deficits resulting from both the stenotic process and the surgery itself. Although Wang et al. specifically studied patients who underwent a specific T-implantation approach, it is logical to infer that implementing a targeted rehabilitation course would be beneficial regardless of the surgical approach used to correct stenotic airways [18]. Further research should evaluate how different laryngotracheal reconstruction techniques might best integrate and benefit from rehabilitation protocols, using standardized outcome measures to compare efficacy across procedures.

Additionally, it is also important to highlight the differences in participant numbers across the studies (Table 2). Schweiger et al. had the largest sample size with 108 patients, while Davis et al. had the smallest, with only nine (Table 2) [20,22]. The disparity in patient numbers introduces potential bias and may affect the internal validity of the analysis of surgical outcomes. To improve the reliability of conclusions, future studies should aim to recruit larger and more balanced cohorts. This would help produce more robust findings and enable stronger, evidence-based recommendations regarding which LTR approach yields the best surgical outcomes.

## Conclusions

Age, sex, and ethnicity were not identified as significant factors in the reviewed studies, suggesting they do not contribute to the risk of requiring laryngotracheal reconstruction. However, the underlying etiology for undergoing LTR was found to be a significant and variable factor, indicating that the cause of the condition plays a crucial role in determining the need for the procedure. This etiological importance underscores the benefit of tailoring the LTR approach to the specific cause of stenosis, potentially enhancing patient outcomes. In addition, rehabilitation appears to be a key component in restoring function and alleviating dysphagia. From our analysis, laryngotracheal reconstruction should be approached within a comprehensive, multimodal treatment framework, and preventive strategies to reduce stenosis represent a logical and vital discussion for otolaryngologists to have with their patients.
